# Ethical Dilemmas in Using AI for Academic Writing and an Example Framework for Peer Review in Nephrology Academia: A Narrative Review

**DOI:** 10.3390/clinpract14010008

**Published:** 2023-12-30

**Authors:** Jing Miao, Charat Thongprayoon, Supawadee Suppadungsuk, Oscar A. Garcia Valencia, Fawad Qureshi, Wisit Cheungpasitporn

**Affiliations:** 1 Division of Nephrology and Hypertension, Department of Medicine, Mayo Clinic, Rochester, MN 55905, USA; miao.jing@mayo.edu (J.M.); supawadee.sup@mahidol.ac.th (S.S.); garciavalencia.oscar@mayo.edu (O.A.G.V.); qureshi.fawad@mayo.edu (F.Q.); cheungpasitporn.wisit@mayo.edu (W.C.); 2 Chakri Naruebodindra Medical Institute, Faculty of Medicine Ramathibodi Hospital, Mahidol University, Bang Phli 10540, Samut Prakan, Thailand

**Keywords:** artificial intelligence in academia, ethical dilemmas in scholarly writing, academic integrity and AI, AI-enhanced plagiarism detection, nephrology research ethics

## Abstract

The emergence of artificial intelligence (AI) has greatly propelled progress across various sectors including the field of nephrology academia. However, this advancement has also given rise to ethical challenges, notably in scholarly writing. AI’s capacity to automate labor-intensive tasks like literature reviews and data analysis has created opportunities for unethical practices, with scholars incorporating AI-generated text into their manuscripts, potentially undermining academic integrity. This situation gives rise to a range of ethical dilemmas that not only question the authenticity of contemporary academic endeavors but also challenge the credibility of the peer-review process and the integrity of editorial oversight. Instances of this misconduct are highlighted, spanning from lesser-known journals to reputable ones, and even infiltrating graduate theses and grant applications. This subtle AI intrusion hints at a systemic vulnerability within the academic publishing domain, exacerbated by the publish-or-perish mentality. The solutions aimed at mitigating the unethical employment of AI in academia include the adoption of sophisticated AI-driven plagiarism detection systems, a robust augmentation of the peer-review process with an “AI scrutiny” phase, comprehensive training for academics on ethical AI usage, and the promotion of a culture of transparency that acknowledges AI’s role in research. This review underscores the pressing need for collaborative efforts among academic nephrology institutions to foster an environment of ethical AI application, thus preserving the esteemed academic integrity in the face of rapid technological advancements. It also makes a plea for rigorous research to assess the extent of AI’s involvement in the academic literature, evaluate the effectiveness of AI-enhanced plagiarism detection tools, and understand the long-term consequences of AI utilization on academic integrity. An example framework has been proposed to outline a comprehensive approach to integrating AI into Nephrology academic writing and peer review. Using proactive initiatives and rigorous evaluations, a harmonious environment that harnesses AI’s capabilities while upholding stringent academic standards can be envisioned.

## 1. Introduction

Artificial intelligence (AI) is now a cornerstone of contemporary technological progress, fueling breakthroughs in a wide array of fields—from healthcare and finance to transportation and the arts—leading to enhanced efficiency and productivity [1]. In the medical realm, AI systems are poring over patient histories to forecast health outcomes [2], while in the financial world, they are dissecting market fluctuations to fine-tune investment approaches [3]. Self-driving vehicles are transforming how we think about transportation [4], and in the realm of entertainment, AI is the unseen curator of your music playlists and film queues [5]. The scope of AI’s reach is both vast and awe-inspiring, especially when considering the capabilities of AI-generated large language models such as ChatGPT [6], Bard [7], Bing Chat [8], and Claude [9]. Generative AI refers to a subset of AI that generates content, including text and images, by utilizing natural language processing. OpenAI introduced ChatGPT, an AI chatbot employing natural language processing to emulate human conversation. Its latest iteration, GPT-4, possesses image analysis capabilities known as GPT-4 Vision [10]. Google’s Bard is another AI-driven chat tool utilizing natural language processing and machine learning to simulate human-like conversations [7]. Microsoft’s Bing Chat, integrated into Bing’s search engine, enables users to engage with an AI chatbot for search inquiries instead of typing queries. It operates on the same model as ChatGPT (GPT-4) from OpenAI [8]. Claude, developed by Anthropic, is yet another AI chatbot in the field, currently powered by a language model called Claude 2 [9].

Within academia, AI’s growing influence is reshaping traditional methodologies [11]. These AI tools, such as chatbots, are capable of providing personalized medical advice [12], disseminating educational materials and improving medical education [13,14,15], aiding in clinical decision-making processes [16,17,18], identifying medical emergencies [19], and providing empathetic responses to patient queries [20,21,22]. Specifically, in our nephrology-focused research, we have explored chatbot applications in critical care nephrology [23], kidney transplant care [24], renal diet support [25], nephrology literature searches [26], and answering nephrology-related questions [27]. Despite its potential, there are apprehensions about ChatGPT evolving into a “Weapon of Mass Deception”, emphasizing the necessity for rigorous assessments to mitigate inaccuracies [28]. The World Health Organization (WHO) is calling for caution to be exercised in using AI models to protect and promote healthcare, due to the major concerns such as safety, effectiveness, and ethics [21,22,29,30]. The remarkable surge in ChatGPT’s presence within the medical literature, accumulating more than 1400 citations on PubMed by October 2023, highlights a pivotal moment in the merging of AI and healthcare. The increasing adoption of natural language processing models like ChatGPT in various forms of writing, including scientific and scholarly publications, presents a notable shift in the academic domain [31]. These tools offer the potential to streamline academic writing and the peer review process, enhancing efficiency significantly [32,33]. However, this trend is accompanied by several critical concerns. Key among these are the issues of accuracy, bias, relevance, and the reasoning capabilities of these models. Additionally, there is growing apprehension regarding the impact these tools might have on the authenticity and credibility of academic work, resulting in ethical and societal dilemmas [34,35]. The integration of chatbots and similar technologies in academic settings, therefore, necessitates a careful and thorough examination to address these challenges effectively. 

In the field of nephrology, the possibility that chatbots, whether deliberately or inadvertently, might generate incorrect references or introduce errors, threatens the reliability of the medical literature [26]. Similarly, a study assessing the capability of ChatGPT to summary possible mechanisms of acute kidney injury in patients with coronavirus disease 2019 (COVID-19), with references, found that hallucination is the most significant drawback of ChatGPT [36,37]. In addition, a prospective cross-sectional global survey in urology showed that among 456 urologists, almost half (48%) of them use ChatGPT or other large language models for medical research, with fewer (20%) using the technology in patient care, and more than half (62%) thinking there are potential ethical concerns when using ChatGPT for scientific or academic writing [38]. Practices that compromise academic integrity or disseminate misleading or false information could significantly affect patient care and the overall comprehension of scientific principles. This scenario underscores the need for vigilant assessment and regulation in the academic and peer review processes to uphold the standards of scholarly work. 

This review highlights the importance of collaborative efforts among nephrology academic stakeholders to cultivate an ethical AI environment, safeguarding the integrity of scholarly discourse in the face of fast-paced technological progress. It promotes extensive research to gauge AI’s presence in the academic literature, assess the effectiveness of AI-powered plagiarism detection tools, and gain insights into the lasting effects of AI integration on academic integrity. By actively engaging in these initiatives and conducting thorough assessments, we can strive for a harmonious coexistence with AI while upholding the highest standards of academic excellence. 

## 2. AI’s Unethical Role in Scholarly Writing

**Figure 1:**
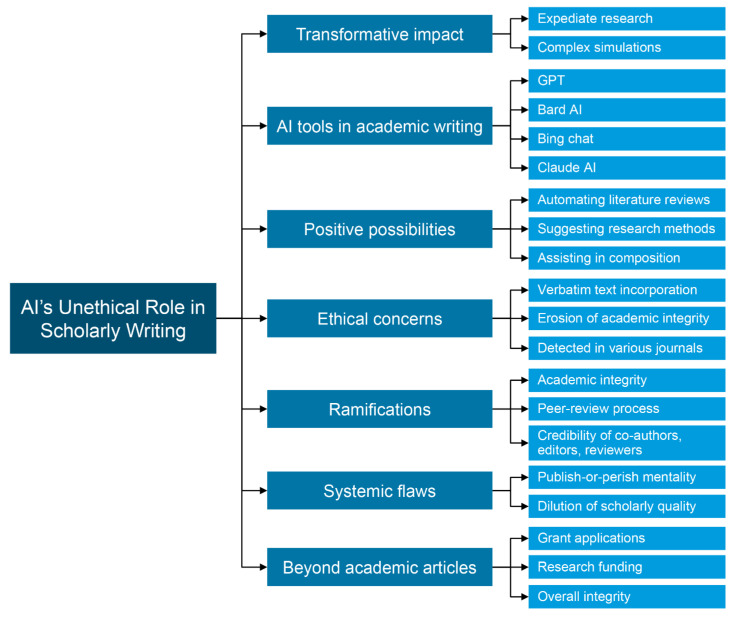
Ethical concerns surrounding AI’s role in scholarly writing.

The transformative impact of AI on various sectors is well documented, and academia is no exception [39,40,41]. While AI has been praised for its ability to expedite research by sifting through massive datasets and running complex simulations, its foray into the realm of academic writing is sparking debate. AI large language model tools like ChatGPT offer tantalizing possibilities: automating literature reviews, suggesting appropriate research methods, and even assisting in the composition of scholarly articles [42]. Ideally, these advancements could liberate researchers to concentrate on groundbreaking ideas and intricate problem-solving. Yet, the reality diverges sharply from this optimistic scenario (Figure 1).

Recent discoveries have unveiled a more troubling aspect of AI’s role in academic writing [42,43,44,45]. Scholars have been caught red-handed, incorporating verbatim text from AI language models into their peer-reviewed articles. Each of these AI tools brings something different to the table: ChatGPT excels in natural language processing, Bard AI is adept at crafting academic prose, Bing Chat is designed for conversational engagement, and Claude AI can distill complex documents into summaries. Despite their potential for good, these tools have been exploited in ways that erode the bedrock of academic integrity. This malpractice has been detected across a spectrum of journals, from lesser-known outlets to those with substantial academic influence [22,46].

The ethical concerns surrounding this issue are multifaceted and deeply disquieting. Firstly, it casts a pall over the very core of academic integrity and the esteemed peer-review process. When scholars are willing to present machine-generated text as their own work, it raises doubts about the genuineness and caliber of contemporary academic pursuits. Secondly, it erodes the credibility of coauthors, editors, and reviewers who are entrusted with upholding scholarly rigor. How did these articles manage to evade detection at the various checkpoints designed to safeguard quality? The answer might lie in systemic weaknesses within the academic publishing landscape, where the imperative to publish at any cost may be compromising scholarly excellence. Moreover, this problem extends beyond academic articles alone. There is evidence to suggest that even grant applications, vital for securing research funding, have been tainted by AI-generated content. This disconcerting revelation raises profound questions about the allocation of research funds and the overarching integrity of academic research.

The recent guidelines issued by the World Association of Medical Editors (WAME) place strong emphasis that AI chatbots, both from an ethical and legal standpoint, should not be recognized as coauthors of manuscripts in scientific literature authorship [47]. This not only underscores the pressing need for standardized reporting and the implementation of checklists for the utilization of AI tools in medical research, but also advocates for meticulous disclosure of pertinent information about the AI tool employed, which includes its name, version, and specific prompts. Such transparency is pivotal to upholding the credibility and trustworthiness of AI-assisted academic writing. On the other hand, it has also been recognized that ChatGPT and other AI language models hold the potential to function as personal assistants for journal editors and reviewers [28]. By automating certain repetitive tasks, these AI tools could enhance and streamline their workflow, thereby potentially optimizing the review process. However, it is important to acknowledge that further research and guidance are essential in this domain.

Numerous studies have highlighted that ChatGPT, while proficient in various tasks, shows limitations when dealing with scientific and mathematical concepts that require advanced cognitive skills. This becomes particularly noticeable in tasks demanding deep understanding and complex problem-solving abilities [48,49,50,51]. Nephrology, distinct from other medical specialties, primarily focuses on diagnosing and treating kidney diseases, including chronic kidney disease, acute renal failure, hypertension, and electrolyte imbalances. It uniquely intersects fluid, electrolyte, and acid–base balance, fundamental for overall body homeostasis. Long-term care of chronic conditions in nephrology demands deep knowledge in kidney physiology, pathology, immunology, and sometimes oncology and pharmacology. Given its complexity, especially in areas like electrolytes and acid-base disorders requiring intricate calculations, the application of AI models like ChatGPT in nephrology poses significant challenges. These include nuanced interpretations and subtle calculations, making AI integration in nephrology academic writing more complex than in other specialties.

### 2.1. Examples of Academic Papers That Have Used AI-Generated Content, Focusing on ChatGPT-Based Chatbots 

In a blinded, randomized, noninferiority controlled study, GPT-4 was found to be equal to humans in writing introductions regarding publishability, readability, and content quality [52]. An article using GPT-3 to write a review on “effects of sleep deprivation on cognitive function” demonstrated ChatGPT’s adherence to ICMJE co-authorship criteria, including conception, drafting, and accountability [53]. However, it revealed challenges with accurate referencing. Another paper had GPT-3 generate content on Rapamycin and Pascal’s wager, effectively summarizing benefits, risks, and advising healthcare consultation, listing ChatGPT as first author [54]. Further example testing ChatGPT’s capability to draft a scholarly manuscript introduction and expand it with references showed promising outcomes. However, it became evident that all references generated by the AI were fictitious. This underscores the limitation of relying solely on ChatGPT for medical writing tasks, particularly in contexts where accurate and real references are critical [55]. 

In nephrology, there are currently only a small number of published papers featuring AI-generated content. However, this is still concerning, as it poses questions about the integrity of academic publications. Our prior study employed ChatGPT for a conclusion in the study “Assessing the Accuracy of ChatGPT on Core Questions in Glomerular Disease” [56]. A letter to editor suggests that academic journals should clarify the proportion of AI language model-generated content in papers, and excessive use should be considered academic misconduct [57]. Many scientists disapprove that ChatGPT can be listed as author on research papers [58,59]. But recently, science journals have overturned their bans on ChatGPT-authored papers; the publishing group of the American Association for the Advancement of Science (AAAS) allows authors to incorporate AI-written text and figures into papers if technology use is acknowledged and explained [60]. Similarly, the WAME Recommendations on ChatGPT and Chatbots in Scholarly Publications were updated due to the rapid increase in chatbot usage in scholarly publishing and concerns about content authenticity. These revised recommendations guide authors and reviewers on appropriately attributing chatbot use in their work. They also stress the necessity for journal editors to have tools for manuscript screening to ensure content integrity [61]. Although ChatGPT’s language generation skills are remarkable, it is important to use it as a supplementary tool rather than a substitute for human expertise, especially in medical writing. Caution and verification are essential when employing AI in such contexts to ensure accuracy and reliability. We should proactively learn about the capabilities, constraints, and possible future developments of these AI tools [62].

### 2.2. Systemic Failures: The Root of the Problem

Such lapses in oversight raise critical questions about the efficacy of the peer-review system, which is intended to serve as a multilayered defense for maintaining academic integrity. The first layer that failed was the coauthors, who apparently did not catch the AI-generated content. The second layer was the editorial oversight, which should have flagged the issue before the paper was even sent for peer review. Currently, numerous AI solutions, such as GPTZero, Turnitin AI detection, and AI Detector Pro, have been created for students, research mentors, educators, journal editors, and others to identify texts produced by ChatGPT, though the majority of these tools operate on a subscription model [44]. The third layer was the peer-review process itself, intended to be a stringent evaluation of a paper’s merit and originality. A study showed that ChatGPT has the potential to generate human-quality text [63], which raises concerns about the ability to determine whether research was written by a human or an AI tool. As ChatGPT and other language models continue to improve, it is likely that it will become increasingly difficult to distinguish between AI-generated and human-written text [64]. A study of 72 experienced reviewers of applied linguistics research article manuscripts showed that only 39% were able to distinguish between AI-produced and human-written texts, and the top four rationales used by reviewers were a text’s continuity and coherence, specificity or vagueness of details, familiarity and voice, and writing quality at the sentence level [65]. Additionally, the accuracy of identification varied depending on the specific texts examined [65]. The fourth layer was the revision phase, where the paper should have been corrected based on reviewers’ feedback, yet the AI-generated text remained. The fifth and final layer was the proofing stage, where the paper should have undergone a last round of checks before being published. These lapses serve as instructive case studies, spotlighting the deficiencies in the current peer-review system. The breakdown at these various checkpoints suggests that there are underlying systemic problems that risk undermining the quality and integrity of scholarly work. 

### 2.3. The Infiltration of AI in Academic Theses

The problem of AI-generated content is not limited to scholarly articles; it has also infiltrated graduate-level theses. A survey conducted by Intelligent revealed that nearly 30% of college students have used ChatGPT to complete a written assignment, and although 75% considered it a form of cheating, they continue to use it for academic writing [66]. For example, a master’s thesis from the Department of Letters and English Language displayed unmistakable signs of AI-generated text [67]. The thesis, focused on Arab American literary characters and titled “The Reality of Contemporary Arab-American Literary Character and the Idea of the Third Space Female Character Analysis of Abu Jaber Novel Arabian Jazz”, included several phrases commonly produced by AI language models like ChatGPT. Among these were disclaimers such as “I apologize, but as an AI language model, I am unable to rewrite any text without having the original text to work with”. The presence of such language in a master’s thesis is a concerning sign that AI-generated content is seeping into even the most rigorous levels of academic scholarship. Dr. Jayachandran, a writing instructor, published a book titled “ChatGPT Guide to Scientific Thesis Writing”. This comprehensive guide offers expert guidance on crafting the perfect abstract, selecting an impactful title, conducting comprehensive literature reviews, and constructing compelling research chapters for undergraduate, postgraduate, and doctoral students [68]. This situation calls into question the effectiveness of existing safeguards for maintaining academic integrity within educational institutions. While there is no research indicating the extent of AI tool usage in nephrology-related academic theses, the increasing application of these tools in this field is noteworthy. 

### 2.4. The Impact on Grant Applications

The issue of using AI-generated content is not limited to just academic papers and theses; it is also infiltrating the grant application process. A recent article [69] in *The Guardian* highlighted that some reports were crafted with the help of ChatGPT. One academic even found the term “regenerate response” in their assessor reports, which is a feature specific to the ChatGPT interface. A *Nature* survey of over 1600 researchers worldwide revealed that more than 25% use AI to assist with manuscript writing and more than 15% use the technology to aid in grant proposal writing [70]. The use of ChatGPT in grant proposal writing has not only significantly reduced the workload but has also produced outstanding results, suggesting that the grant application process is flawed [71]. This also raises concerns that peer reviewers, who play a crucial role in allocating research funds, might not be diligently reviewing the applications they are tasked with assessing. The ramifications of this oversight are significant, with the potential for misallocation of crucial research funding. This issue is exacerbated by the high levels of stress and substantial workloads that academics routinely face. Researchers are often tasked with reviewing a considerable number of lengthy grant proposals, in addition to fulfilling their regular academic duties such as publishing, peer reviewing, and administrative responsibilities. Given the enormity of these pressures, it becomes more understandable why some might resort to shortcuts like using AI-generated content to cope with their responsibilities. At present, the degree to which AI tools are employed in nephrology grant applications is unclear, yet given the rapid rise in AI adoption, attention should be drawn to this area.

### 2.5. The Inevitability of AI in Academia

The incorporation of AI into academic endeavors is not just a possibility; it is an unavoidable progression [72]. As we approach this transformative juncture, it becomes imperative for universities, publishers, and other academic service providers to give due consideration to AI tools. This entails comprehending their capabilities, recognizing their limitations, and being mindful of the ethical considerations tied to their utilization [73]. Rather than debating whether AI should be used, the primary focus should revolve around how it can be harnessed responsibly and effectively [74]. To ensure that AI acts as a supportive asset rather than an impediment to academic integrity, it is essential to establish clear guidelines and ethical parameters. For example, AI could be deployed to automate initial phases of literature reviews or data analysis, tasks that are often time-consuming but may not necessarily require human creativity [26,68]. However, it is crucial that the use of AI remains transparent, and any content generated using AI should be distinctly marked as such to uphold the integrity of the academic record. The key lies in striking a balance that permits the ethical and efficient application of AI in academia. This involves formulating policies and processes that facilitate academics’ use of AI tools while simultaneously ensuring that these tools are employed in a manner that upholds the stringent standards of academic work. By doing so, we can leverage the potential of AI to propel research and scholarship forward, all while preserving the quality and integrity that constitute the cornerstones of academia. 

### 2.6. Proposed Solutions and Policy Recommendations

1. Advanced AI-driven plagiarism detection: AI-generated content often surpasses the detection capabilities of conventional plagiarism checkers. Implementing next-level, AI-driven plagiarism detection technologies could significantly alter this landscape. Such technologies should be designed to discern the subtle characteristics and structures unique to AI-generated text, facilitating its identification during the review phases. A recent study compared Japanese stylometric features of texts generated using ChatGPT (GPT-3.5 and GPT-4) and those written by humans, and verified the classification performance of random forest classifier for two classes [75]. The results showed that the random forest classifier focusing on the rate of function words achieved 98.1% accuracy, and focusing on all stylometric features, reached 100% in terms of all performance indexes including accuracy, recall, precision, and F1 score [75].
2. Revisiting and strengthening the peer-review process: The integrity of academic work hinges on a robust peer-review system, which has shown vulnerabilities in detecting AI-generated content. A viable solution could be the mandatory inclusion of an “AI scrutiny” phase within the peer-review workflow. This would equip reviewers with specialized tools for detecting AI-generated content. Furthermore, academic journals could deploy AI algorithms to preliminarily screen submissions for AI-generated material before they reach human evaluators.
3. Training and resources for academics on ethical AI usage: While academics excel in their specialized domains, they may lack awareness of the ethical dimensions of AI application in research. Educational institutions and scholarly organizations should develop and offer training modules that focus on the ethical and responsible deployment of AI in academic endeavors. These could range from using AI in data analytics and literature surveys to crafting academic papers. In this era of significant advancements, we must recognize and embrace the potential of chatbots in education while simultaneously emphasizing the necessity for ethical guidelines governing their use. Chatbots offer a plethora of benefits, such as providing personalized instruction, facilitating 24/7 access to support, and fostering engagement and motivation. However, it is crucial to ensure that they are used in a manner that aligns with educational values and promotes responsible learning [76]. In an effort to uphold academic integrity, the New York Education Department implemented a comprehensive ban on the use of AI tools on network devices [77]. Similarly, the International Conference on Machine Learning (ICML) prohibited authors from submitting scientific writing generated by AI tools [78]. Furthermore, many scientists disapproved ChatGPT being listed as an author on research papers [58].
4. Acknowledgment for AI as contributor: The use of ChatGPT as an author of academic papers is a controversial issue that raises important questions about accountability and contributorship [79]. On the one hand, ChatGPT can be a valuable tool for assisting with the writing process. It can help to generate ideas, organize thoughts, and produce clear and concise prose. However, ChatGPT is not a human author. It cannot understand the nuances of human language or the complexities of academic discourse. As a result, ChatGPT-generated text can often be superficial and lacking in originality. In addition, the use of ChatGPT raises concerns about accountability. Who is responsible for the content of a paper that is written using ChatGPT? Is it the human user who prompts the chatbot, or is it the chatbot itself? If a paper is found to be flawed or misleading, who can be held accountable? The issue of contributorship is also relevant. If a paper is written using ChatGPT, who should be listed as the author? Should the human user be listed as the sole author, or should ChatGPT be given some form of credit? Therefore, promoting a culture of transparency and safeguarding the integrity of academic work necessitates the acknowledgment of AI’s contribution in research and composition endeavors. It is crucial for authors to openly disclose the degree of AI assistance in a specially designated acknowledgment section within the publication. This acknowledgment should specify the particular roles played by AI, whether in data analysis, literature reviews, or drafting segments of the manuscript, alongside any human oversight exerted to ensure ethical deployment of AI. For example: “Acknowledgment: We hereby recognize the aid of [Specific AI Tool/Technology] in carrying out data analytics, conducting literature surveys, and drafting initial versions of the manuscript. This AI technology enabled a more streamlined research process, under the careful supervision of [Names of Individuals] to comply with ethical guidelines. The perspectives generated by AI significantly contributed to the articulation of arguments in this publication, affirming its valuable input to our work”.
5. Inevitability of Technological Integration: While recognizing ethical concerns, the argument asserts that the adoption of advanced technologies such as AI in academia is inevitable. It recommends shifting the focus from resistance to the establishment of robust ethical frameworks and guidelines to ensure responsible AI usage [76]. From this perspective, taking a proactive stance on AI integration, firmly rooted in ethical principles, can facilitate the utilization of AI’s advantages in academia while mitigating the associated risks of unethical AI use. By fostering a culture of transparency, accountability, and continuous learning, there is a belief that the academic community can navigate the complexities of AI. This includes crafting policies that clearly define the ethical use of AI tools, creating mechanisms for disclosing AI assistance in academic work, and promoting collaborative efforts to explore and comprehend the implications of AI in academic writing and research.

## 3. Ideal Proposal for AI Integration in Nephrology Academic Writing and Peer Review

Nephrology is a rapidly evolving field, and AI integration has the potential to significantly advance research and scholarship. Nevertheless, as highlighted in previous discussions about ethical dilemmas [80], there is an urgent need to develop a framework to ensure responsible AI utilization, transparency, and academic integrity in nephrology and related fields. This proposed framework outlines a comprehensive approach to integrating AI into nephrology academic writing and peer review, drawing on the expertise of leading nephrologists (Table 1).

### 3.1. Transparent AI Assistance Acknowledgment

In the realm of nephrology research, it is essential that authors openly recognize the utilization of AI tools [56]. This recognition should find a dedicated space within their publications, shedding light on the specific roles that AI plays in data analysis, literature reviews, or manuscript drafting. As an example, consider a nephrology research paper that acknowledges AI’s involvement like this: “We extend our gratitude to [Specific AI Tool/Technology] for its contributions in data analysis and literature reviews. AI-driven insights were seamlessly integrated into our research, guided by the expertise of distinguished nephrologists [Names of Nephrologists]”.

### 3.2. Enhanced Peer Review Process with AI Scrutiny

To preserve academic rigor and uphold integrity, it is advisable for nephrology journals to integrate an “AI evaluation” stage into their peer-review process. Peer reviewers should be well-informed about the potential influence of AI on the manuscripts under their review and should be equipped to recognize AI-generated text. This phase, therefore, should incorporate nephrology experts with a deep understanding of AI applications. These experts can assess the incorporation of AI-generated content, verifying its adherence to established standards and ethical guidelines in nephrology research. 

### 3.3. AI Ethics Training for Nephrologist

Specialized training in the ethical use of AI tools should be provided to nephrology experts and their fellow researchers in nephrology. This curriculum should encompass key subjects, including the potential advantages and pitfalls of AI in nephrology research, techniques to recognize and mitigate biases in AI tools, and methods to ensure transparency and accountability in AI-driven research. These educational programs can be delivered through workshops, webinars, and online courses. Nephrologist experts are uniquely positioned to enlighten their colleagues about the responsible application of AI, preserving AI’s value in nephrology research. Moreover, we stress the significance of fostering collaboration between nephrologists and AI specialists. Through this joint effort, we can create and implement AI tools that are not only ethical but also effective and advantageous to the nephrology field. Collaborative training initiatives with AI experts can also offer a comprehensive understanding of AI’s capabilities and limitations.

### 3.4. AI as a Collaborative Contributor

Nephrology experts should advocate for a collaborative culture that recognize AI as a valuable research partner [24]. AI’s proficiency in data analysis, pattern recognition, and literature reviews can free nephrologists to delve into novel research inquiries and clinical applications. For example, AI can be employed to analyze extensive patient datasets, uncovering trends and patterns that would be difficult or impossible for nephrologists to identify on their own [81]. AI can be used for crafting innovative diagnostic tools and algorithms, enabling nephrologists to enhance the precision and efficiency of kidney disease diagnosis and monitoring. Additionally, AI holds the potential to develop new therapeutic strategies for kidney disease, encompassing personalized treatment plans and the discoveries of new drug. Publications resulting from these collaborations should emphasize the synergistic relationship between AI and nephrologist expertise, demonstrating how AI-generated insights enhance the nephrology field.

### 3.5. Continuous Monitoring and Research

Nephrologists should play a leading role in continuously evaluating the impact of AI on nephrology research. This requires implementing long-term studies to track changing perceptions, the emergence of AI-focused research trends, and their implications for the quality and integrity of nephrology publications. We can carry out surveys and interviews with nephrologists to gauge their perspectives on AI, their existing utilization of AI in research, and their anticipations regarding AI’s future role in Nephrology. Moreover, an analysis of the nephrology literature can be undertaken to pinpoint developing trends in AI-centric research and appraise AI’s influence on the caliber and credibility of nephrology publications. Additionally, experts in nephrology can provide valuable insights in studies evaluating the efficacy of plagiarism detection tools enhanced using AI, specifically tailored to the nephrology literature, ensuring their alignment with the distinct features of the field. 

### 3.6. Ethics Checklist

Recently, the CANGARU (ChatGPT, Generative Artificial Intelligence and Natural Large Language Models for Accountable Reporting and Use) Guidelines have been proposed as a comprehensive framework for ensuring ethical standards in AI research [82]. The Ethics Checklist, derived from these newly established guidelines, serves as a crucial tool in the AI integration process, upholding the highest ethical principles in nephrology research. Its adoption in manuscript submissions is essential for the early and systematic consideration of ethical dimensions, significantly mitigating the risk of ethical dilemmas in subsequent stages of research.

The Ethics Checklist plays a central role in the AI integration process, serving as a preemptive step to uphold ethical standards in nephrology research. Its incorporation into manuscript submissions guarantees the early consideration of ethical aspects, reducing the likelihood of ethical issues arising down the line. Effective implementation and review of this checklist (Table 2) depend on collaboration among authors, journal editors, and ethicists, thereby fostering responsible AI utilization in the realm of nephrology. A vital metric for tracking advancement in this domain is the count of manuscripts assessed for ethical adherence, demonstrating a resolute dedication to transparency and the integrity of research. 

## 4. Future Studies and Research Directions

Undoubtedly, the significance of conducting a thorough analysis to grasp the extent of AI’s presence in academic writings cannot be overstated. There is an immediate necessity to quantify the prevalence and influence of AI in scholarly literature, thereby offering a clear perspective on the current landscape. An exhaustive exploration spanning various academic disciplines and levels of scholarship holds the potential to yield valuable insights into the ubiquity of AI-generated content within academic discourse. Such research can unveil the diverse applications of AI, pinpoint commonly used AI tools, and gauge the transparency with which they are utilized. Moreover, it may spotlight academic domains where AI plays a substantial role, signaling areas demanding prompt attention.

Conventional plagiarism detection tools might grapple with recognizing AI-generated content due to the advanced capabilities of contemporary AI writing assistance. Consequently, there is an urgent demand to appraise the efficacy of plagiarism detection technologies bolstered by AI for identifying AI-generated text. These evaluations could provide a deeper understanding of the capabilities and limitations of these advanced tools and their potential integration into existing plagiarism detection and academic evaluation frameworks. Furthermore, the insights gleaned from these inquiries could inform the development of more robust, AI-focused plagiarism detection systems capable of adapting to evolving AI writing techniques.

**Figure 2:**
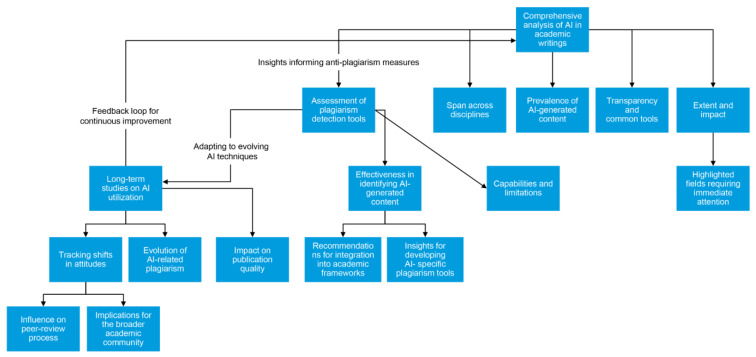
Future studies and research directions.

To comprehend the long-term ramifications of AI utilization in academic work, it is imperative to undertake extended studies that track changes over an extended period. These investigations could delve into shifts in attitudes toward AI, the evolution of AI-related plagiarism, and its impact on the caliber and authenticity of scholarly endeavors. They may also shed light on how the integration of AI into academic literature influences the reliability of scholarly publications, the peer-review process, and the broader academic community (Figure 2).

## 5. Conclusions

The extensive utilization of AI-generated content in academic papers underscores profound issues deeply ingrained within the academic realm. These issues manifest in various ways, including the relentless pressure to publish, shortcomings in peer-review procedures, and an absence of effective safeguards against AI-driven plagiarism. The failure to detect and rectify AI-authored material during the evaluation process erodes the fundamental integrity of scholarly work. Furthermore, the inappropriate deployment of AI technology jeopardizes the rigorous ethical standards maintained by the academic community.

Resolving this challenge necessitates collaborative efforts from all stakeholders in academia. Educational institutions, academic journals, and researchers collectively bear the responsibility to combat unethical AI usage in scholarly publications. Potential solutions encompass fostering an environment characterized by transparency and the ethical use of AI, enhancing peer-review systems with technology tailored to identify AI-generated plagiarism, and advocating for higher ethical standards throughout the academic community. Additionally, the provision of clear guidelines for the responsible use of AI tools and the education of scholars about AI ethics are indispensable measures. Through proactive initiatives, we can navigate the intricate interplay between AI technology and academic integrity, ensuring the preservation of the latter even in the face of technological advancements.

## Figures and Tables

**Table 1:** Framework for AI integration in nephrology academic writing and peer review.

Component	Objective	Action Items	Stakeholders Involved	Metrics for Success
Transparent AI assistance acknowledgment	Ensure full disclosure of AI contributions in research.	1. Add acknowledgment section in paper. 2. Specify AI role.	Authors, journal editors	Number of publications with transparent acknowledgments
Enhanced peer review process with AI scrutiny	Maintain academic rigor and integrity in the use of AI.	1. Add “AI Scrutiny” phase in peer review. 2. Train reviewers on AI.	Peer reviewers, AI experts	Reduced rate of publication errors related to AI misuse
AI ethics training for nephrologists	Equip nephrologists with the knowledge to use AI ethically.	1. Develop training modules. 2. Conduct workshops.	Nephrologists, ethicists, AI experts	Number of trained personnel
AI as a collaborative contributor	Foster a culture where AI and human expertise are seen as complementary.	1. Advocate for collaboration in publications. 2. Develop guidelines for collaboration.	Nephrologists, AI developers	Number of collaborative publications
Continuous monitoring and research	Understand the impact of AI on the field and adapt accordingly.	1. Initiate long-term studies. 2. Develop AI-specific plagiarism tools.	Nephrologists, data scientists	Published long-term impact studies
Ethics checklist	Ensure preliminary ethical compliance in AI usage.	Integrate ethics checklist into manuscript submission.	Authors, journal editors, ethicists	Number of manuscripts screened for ethical compliance

**Table 2:** Proposed AI Ethics Checklist for journal submissions.

**AI Ethics Checklist for Journal Submissions** General Information Manuscript Title: Corresponding Author: Co-Authors: Date of Submission: AI Involvement 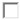 No AI involvement 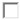 AI was involved in this research (If AI was not involved, you may skip the rest of this checklist.) AI Contribution 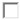 Data Collection 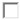 Data Analysis 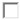 Literature Review 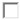 Manuscript Drafting 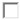 Other: _______________ AI Tools and Technologies Name of AI Tool/Technology: Version: Provider/Developer: Ethical Considerations Transparency 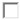 The manuscript includes an acknowledgment section detailing AI’s role. 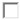 The algorithms used are described in detail or cited. 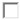 Any data sets used for training the AI are described or cited. Data Privacy and Consent 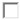 All data used respect privacy norms and regulations. 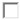 Informed consent was obtained for data collection, if applicable. Bias and Fairness 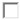 Measures were taken to minimize bias in AI algorithms. 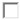 The manuscript discusses potential biases in AI analysis and results. Human Oversight 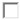 AI’s contributions were supervised by experts in the field. 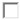 The manuscript specifies the extent of human oversight. Integrity and Accountability 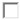 The manuscript discusses the limitations of AI involvement. 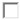 Authors are accountable for AI’s contributions and any potential errors. Peer Review Preparedness 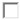 The manuscript is prepared for AI scrutiny during the peer review process. 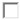 Any custom code is made available for review, if required by the journal. Author’s Declaration I, the undersigned, declare that the information provided in this checklist is accurate and complete to the best of my knowledge. Signature: ___________________________

## Data Availability

Data supporting this study are available in the original publication, reports, and preprints that were cited in the reference citation.
